# White American transgender adults' retrospective reports on the social and contextual aspects of their gender identity development

**DOI:** 10.1111/bjdp.12480

**Published:** 2024-03-06

**Authors:** Emily Herry, S. M. Rodan, Madeline Martin, Mariam M. Sanjak, Kelly Lynn Mulvey

**Affiliations:** ^1^ The Ohio State University Columbus Ohio USA; ^2^ The University of North Carolina at Chapel Hill Chapel Hill North Carolina USA; ^3^ North Carolina State University Raleigh North Carolina USA

**Keywords:** context, gender diversity, identity development, interpretive phenomenological analysis, nonbinary, qualitative, transgender

## Abstract

A growing body of research has attended to the experiences of transgender and gender non‐conforming (TGN) youth's gender identity development. However, practical and ethical concerns have impeded our ability to understand the experiences of TGN youth. Thus, the aim of this study was to utilize one‐on‐one semi‐structured interviews to explore White American TGN adults' (*N* = 15) retrospective accounts of their gender identity development in childhood and adolescence. Findings demonstrate considerable heterogeneity in TGN adults' retrospective accounts of their gender identity development. However, TGN adults consistently highlighted the role of social (e.g. friends, family and teachers) and contextual (e.g. online, offline, educational and geographical) factors in their gender identity journeys. This study provides new insight into the role of social and contextual factors in TGN adults' retrospective accounts of their gender identity development, demonstrating the importance of continuing to examine these factors in gender diversity research.

Gender identity is a socially constructed category people use to identify and label themselves and others (Maccoby, 1988). Most children, including many transgender children, start to understand their gender identity around 3 years old (Olson et al., 2015; Siegler et al., 2006). However, the timing, trajectory and pathways to the development of one's gender identity are nuanced and examination of the development of gender identity requires attention to cultural, contextual, social and individual differences that may shape one's experiences. Assuming all individuals experience a similar process of gender identity development may exclude transgender and gender non‐conforming (TGN) people (i.e. people whose gender identity and/or expression does not align with their sex or gender assigned at birth; Meier & Labuski, 2013). TGN individuals raised in environments where gender diversity was not acknowledged may have additional hurdles to overcome as they navigate their own identities. Some work has addressed the role of social (e.g. friends and family) and contextual (e.g. cultural context and geographical location) processes in TGN people's gender identity development (Levitt & Ippolito, 2014; McGuire et al., 2016), yet there is much we still do not understand. Therefore, the purpose of this study is to describe White American transgender adults' retrospective accounts of their gender identity journey and the role of social influences and contexts in TGN people's gender identity development.

## GENDER IDENTITY DEVELOPMENT

People are regularly socialized based on their assigned gender (Bockting, 2008; Egan & Perry, 2001). Many scholars have utilized self‐socialization theories to understand the influences of self‐socialization on children's gender development. For example, Kohlberg's Cognitive Developmental Theory (Kohlberg, 1966), Gender Schema Theory (Martin & Halverson, 1981; Martin & Ruble, 2004) and Dual Identity Theory (Liben & Bigler, 2002) have been used to understand gender identity development. Theories such as these suggest that once a person, typically a child, identifies their gender, they are motivated to understand and learn more about that identity. One study of binary transgender and cisgender children has demonstrated developmental patterns that support this idea; once children acknowledge their gender identity, even if different from their sex assigned at birth, they then begin engaging in gendered learning towards the social norms associated with their gender identity (Gülgöz et al., 2019). The current study adds to this literature by addressing the role that social and contextual factors play in the process of TGN people recognizing and socializing their gender identity.

## TGN IDENTITY DEVELOPMENT

Research on the gender identity development of children who do not conform to societal expectations of their gender (i.e. TGN children) has become an area of great interest (Gülgöz et al., 2019; Hässler et al., 2022). Much of our understanding of gender diversity in development has come from the TransYouth Project, a longitudinal study of socially transitioned transgender children's gender identity development (ages 3–12 years at the start of the project; Olson & Gülgöz, 2018; Olson et al., 2015). This research has been vital to our understanding of gender identity development in transgender youth. For example, one study from this project found transgender children's gender cognition was virtually indistinguishable from cisgender children who shared their gender identity (Olson et al., 2015). Other work from this project demonstrated the importance of self‐socialization in gender identity development among transgender youth (Gülgöz et al., 2019).

Yet, to date, less is known about the experiences of TGN youth who did not socially transition early in childhood (e.g. their gender self‐socialization process), nor about those with nonbinary gender identities. Furthermore, with the ongoing challenges faced by transgender youth (Kraschel et al., 2022) and the changing perspectives surrounding gender diversity within society at large (McLean, 2021; Parker et al., 2022), additional research is needed to expand our understanding of gender identity development within TGN youth. Thus, a goal of this study was to explore early experiences of gender diversity through White American TGN adults' retrospective accounts of their own gender identity journey.

## RETROSPECTIVE ACCOUNTS OF GENDER DIVERSITY

Much of the research on TGN gender identity development is specifically with binary transgender children whose parents were supportive of their identities (Olson et al., 2015; Olson & Gülgöz, 2018). Although important, this type of research may not represent the reality for many TGN children (Minter, 2012; Priest, 2019). Yet, conducting research on TGN identity development with children who have not socially transitioned may introduce potential practical and ethical issues (see Reed, 2023). Practically, recruiting these children may be difficult as they may still be in the process of discovering their identity and be unable to verbalize their experiences (Clark, 2018; Clark et al., 2020; Reed, 2023). Ethically, by seeking out consent to participate in research related to gender development or by participating in research activities related to TGN gender development, there is the potential risk that these children may be outed to people who are not supportive of their TGN identity or face other forms of harm (Martin & Meezan, 2003; Reed, 2023).

One way to address this is to study TGN adults' retrospective accounts of their gender identity development (Morgan & Stevens, 2012). In particular, retrospective qualitative research may allow for a more nuanced understanding of TGN gender identity development (Bradford & Syed, 2019). By giving TGN adults the space to reflect on their gender identity journey, researchers can gain a deeper understanding of the complex interplay of social and contextual factors that shape the process of TGN adults' recognizing their gender identity. Thus, the current research utilizes a qualitative design to address TGN adults' retrospective accounts of gender diversity.

## CURRENT STUDY

As researchers continue to recognize gender identity development as a developmentally complex process (Keener, 2015), scholars have started to consider how social relationships and contexts play a role in gender identity development for TGN people, in particular. Recent work has looked towards the role of family, especially parental, support during important moments in transgender and gender‐expansive youth's gender identity development (Andrzejewski et al., 2021; Hale et al., 2021), as well as self‐socialization in this identification process (Gülgöz et al., 2019). Other research has explored contextual factors in the roles of TGN people's li.e. such as access to health care in the Southeastern United States (Johnson et al., 2020), but less work has attended to the role of context in the process of recognizing and socializing their identities. Additional work is needed to understand the complexity of gender identity development. Thus, the current study utilizes qualitative methods to explore White American TGN adults' retrospective accounts of their gender identity journey with a focus on contextual and social factors.

## METHODS

### Participants

TGN adults (*N* = 15; *M*_age_ = 28.27, Range = 22–41) were recruited through online sampling methods (i.e. posting to online groups and listservs) as well as snowball sampling during the Summer of 2022. Sampling continued until no new themes emerged in the interviews (See Table 1 for additional demographic information).

**TABLE 1:** Demographics of study participants.

Variables	Participants *n* (%)
Gender identity	
Transgender woman	2 (13.33%)
Transgender man	5 (33.33%)
Nonbinary/Genderqueer	6 (40%)
Transmasculine	2 (13.33%)
Sex assigned at birth	
Female	11 (73.33%)
Male	4 (26.66%)
Sexual orientation	
Bi+	11 (73.33%)
Gay/Lesbian	3 (20%)
Unsure	1 (6.67%)
Race/Ethnicity	
White	15 (100%)
Education	
Graduated from High School	3 (20%)
Graduated from College	6 (40%)
Master's degree or equivalent	4 (26.66%)
PhD or equivalent	2 (13.33%)
Region of the United States	
Southeast	6 (40%)
Northeast	1 (6.67%)
Midwest	3 (20%)
West	5 (33.33%)

*Note*: Region was calculated based on zip code information. Bi+ is an umbrella term to encompass attraction to more than one gender, including but not limited identities to such as bisexual or pansexual.

### Interview protocol

The semi‐structured interview protocol was developed to understand how TGN people used online and offline sources and communities to learn about TGN identities and how these experiences related to their own gender identity journey. Interview questions analysed for this study addressed: age of identity realization, process of recognizing identity, context of recognizing identity and how other people shaped the process of recognizing identity (Table 2).

**TABLE 2:** Semi‐structured interview guide questions about gender identity journey.

Can you tell me about your gender identity journey? How old were you when you first realized you were transgender or gender non‐conforming? How did you come to recognize yourself as transgender or gender non‐conforming? Tell me about the process as you started to recognize your identity as transgender or gender non‐conforming and potentially started to share that identity with others. What steps, if any, have you taken to feel affirmed in your gender identity? (e.g. haircut or clothing changes, name change, seeking medical or social support/transition) How have other people shaped your gender identity?

*Note*: Interviewer skipped probing questions (a–e) based on participant's response to the primary question.

### Procedure

Following Institutional Review Board (IRB) approval, but prior to recruiting participants, the researchers worked with a community advisory board (CAB) comprised of TGN community members and people who worked closely with the community (*N* = 5). CAB members were recruited through email. The CAB consisted of clinical and research psychologists, students and TGN community advocates. No CAB members participated in the primary study. With their feedback, the interview protocol was adjusted and resubmitted to the IRB. Following approval, participants were recruited. Participants first completed a screening questionnaire followed by the consent forms if eligible. Interviews consisted of one‐on‐one interviews over Zoom with the first author during 2022. Interviews were recorded and then transcribed verbatim. Interviews lasted between 45 and 90 min and the screener and demographic survey took approximately 7–15 min to complete. Following the interviews, participants were invited to review their interview transcript for accuracy (i.e. member checking). This process took between 30 and 60 min to complete and nine participants completed member checking. Participants were provided a $20 gift card for completing the interview and an additional $10 gift card if they participated in member checking.

### Author positionality

The first author is a nonbinary person who grew up and completed their post‐secondary education in the Southeastern United States. Author one's lived experiences motivate them to investigate the ways in which social and contextual factors impact the daily li.e. health and development of TGN people with the goal of using their research to inform interventions to reduce health disparities and promote the health and development of TGN people across their lifespans.

Author 2 is a transgender nonbinary individual from the Southeastern United States. Their academic background lies in developmental psychology and population health science focusing on relating social factors to overall health. Author 2 approached the present project having previously used online resources as a personal means of accessing, understanding and connecting to LGBT+ culture. Their hope is that research will continue to consider the role of online spaces on the social development of TGN persons.

Author 3 is a White genderqueer person from the Northeastern United States, who moved to the Southeastern United States to continue their education. Their research experience and education lie within social developmental psychology, with a special interest in identity development. Author 3's own identity and background inform their contribution to this research; they are committed to producing an empathetic exploration of the diverse narratives of the TGN community.

Author 4 is a cisgender Arab Muslim woman who is pursuing a bachelor's in psychology in the Southeastern United States, with research experience in social development. Her research interests surround addressing mental health disparities in marginalized populations, especially within the Arab Muslim community. Author 4's experience working with the LGBT+ community in her time as a research assistant motivated her contribution to this project.

The last author is a cisgender White woman who grew up in the Mid‐Atlantic United States. She has taught both at the secondary (high school) and post‐secondary levels in the South for more than 20 years. As a developmental scientist, one of her research interests centers on gender identity development and, in particular, the role of societal norms, stereotypes and expectations on gender identity development.

### Theoretical underpinnings and data analysis

Interpretative phenomenological analysis (IPA; Smith & Shinebourne, 2012) was used as the theoretical underpinnings and analytical technique for this study. IPA operates under three primary theoretical principles. First, IPA follows the assumptions of phenomenology, the philosophical study of consciousness and direct, individual experience untethered from pre‐existing theoretical conceptions (Husserl, 2012). Second, IPA aligns with hermeneutics, the assumption that understanding people's individual experiences is an interpretive process (Schleiermacher, 1998). Hence, as researchers, we try to create meaning out of the participants' understanding of their own experiences (Smith & Osborn, 2015). Third, given that this approach addresses how each participant is unique, cases are examined and themes are created for the individual prior to examining all participants together (idiographic inquiry; Braun & Clarke, 2021; Smith & Osborn, 2015). This particular approach was chosen as prior research has indicated that IPA is especially useful when examining emotionally laden, ambiguous and complex topics (Smith & Osborn, 2015), which is descriptive of TGN adults' retrospective accounts of their gender identity journey.

Data were analysed through the three‐step process typical of IPA (Smith & Shinebourne, 2012). Coders included the first author and a team of six undergraduate research assistants (RAs), two of whom identified as TGN and four who identified as cisgender. For each individual participant, we first conducted a descriptive pass to document what was shared. Next, a linguistic pass to look for unique patterns of speech. Lastly, an interpretive pass to dissect what the participants said. During this process, we noted the initial themes that emerged (i.e. memoing).

Following this, we further constructed and clarified emergent themes and subthemes, categorized them within the research questions, constructed a codebook for ease of coding and discussed convergent and divergent responses. Each participant's response was coded by two RAs. After each RA coded a participant's response independently, they met with their coding partner to compare results (i.e. consensus coding). RAs also met with researchers weekly to discuss any potential issues that arose or points of clarity.

## RESULTS

When discussing their gender identity journey, three categories of themes emerged across participant responses: (1) identity recognition and development (i.e. their transition experience including recognition of their identity); (2) contextual influences on gender identity journey (i.e. how their context, such as their geographical location, influenced their gender identity journey); and (3) social influences on gender identity journey (i.e. how their social environment, such as their friends and family, influenced their gender identity journey). Importantly, many of these themes were integrated, as most participants discussed their journey as a holistic process and thus the themes are inextricably linked. Participant extracts are provided to demonstrate the diversity of experiences within the themes, as well as to support the themes, as recommended by Smith (2011). See Table 3 for individual demographic information including an approximation of when participants felt discomfort with or started questioning their assigned gender, as well as the approximate age participants realized they were TGN.

**TABLE 3:** Participant Individual Information.

	Gender identity	Sex assigned at birth	Signs of TGN identity	Realization of TGN identity	Age	Sexual orientation	Education	Subjective SES
Emma	Transgender Women	Male	College	19	22	Unsure	College	6
Sage	Nonbinary/Genderqueer	Female	College	21	23	Gay/Lesbian	Masters	5
Avery	Nonbinary/Genderqueer	Male	Puberty	28	29	Bi+	Masters	6
Drew	Nonbinary/Genderqueer	Female	Childhood	17	24	Bi+	College	6
Kai	Transmasculine	Female	Childhood	17	30	Bi+	Masters	5
Wren	Nonbinary/Genderqueer	Female	College	20–21	25	Bi+	High School	5
Leo	Transgender Man	Female	Puberty	14	23	Bi+	College	6
Noah	Transgender Man	Female	Childhood	Middle School	31	Gay/Lesbian	PhD	7
Andrew	Transgender Man	Female	Childhood	34	41	Bi+	High School	7
Daniel	Transgender Man	Female	Childhood	27	34	Bi+	College	6
Ash	Nonbinary/Genderqueer	Female	High School	16	23	Bi+	Masters	5
Jack	Transgender Man	Female	Childhood	32	41	Bi+	PhD	7
Quinn	Nonbinary/Genderqueer	Male	Childhood	24–25	26	Bi+	College	3
Alice	Transgender Woman	Male	Young Adulthood	23	28	Gay/Lesbian	High School	4
Spencer	Transmasculine	Female	Childhood	17	24	Bi+	College	9

*Note*: Pseudonyms are used for participant names. Signs of TGN identity approximate when participants felt discomfort with or started questioning their assigned gender. Realization of TGN identity denotes when participants indicated they had the language to describe themselves as their identity. Subjective SES was assessed using The MacArthur Scale of Subjective Social Status (Adler et al., 2000), scores of 1–3 indicate low‐subjective SES, 4–7 middle‐subjective SES and 8–10 high‐subjective SES.

### Identity recognition and development

Participants discussed the process of recognizing themselves as TGN, with a great amount of variability between participants in when and how they came to recognize their identities. First, several participants described knowing or having signs their gender identity did not match their assigned gender very early in their lives (i.e. childhood *n* = 8; 4 transgender men; 2 nonbinary/genderqueer; 2 transmasculi.e. yet there was variety in the age participants verbalized their identity.

I always knew that I wasn't a girl … I wanted to go play baseball with the other boys, and my mom was like ‘Well, you can't go play baseball because you're in a dress’, and I'm like, ‘but I don't want to be in a dress’, and she's like ‘well, but you‐you have to wear a dress, you're a girl’ and I'm like, ‘but I'm not though’, I remember being very young, you know, like four and five, and just being like ‘I'm not a girl’. Why do you keep saying that, like, why does everyone not see what I saw as a small child (Daniel, Transgender Man, 34)?

Other participants indicated they did not recognize their identities during childhood (i.e. identifying during adolescence or adulthood; *n* = 7; 4 nonbinary/genderqueer; 2 transgender women; 1 transgender man). Interestingly, nonbinary/genderqueer participants were more likely to indicate that they were unable to put a name to their identities until adolescence or adulthood compared to binary transgender people. This may be because nonbinary/genderqueer participants were less likely to have access to knowledge about identities outside of the binary until later in their lives. Related to this, many participants indicated that identity recognition was an ongoing process, from recognizing that they did not identify with their assigned gender, to being able to put words or labels onto themselves that best fit their identity. Some noted trying out new names or pronouns with small groups of trusted friends as they started to develop their sense of their gender identity. Others noted that they tried labels, such as genderqueer, but then realized that other labels fit them better. Even when they knew they were not cisgender, they did not always have the words to describe their identity or experience until later in their lives. This was reported by five nonbinary/genderqueer participants and both transmasculine participants.

I don't really like conforming to the narrative of like ‘I've known since I was a kid’. Because, like, I mean I didn't, but I did…I remember like being in the pool with friends and playing mermaids and I wanted to be a merman…I could give you a range of answers [of when I knew I was TGN] so an age where like I could name that I wasn't cis like I had vocabulary to do that, that is probably when I was maybe like 17ish and then in terms of like ‘I didn't have the words but like looking back on it’…I never felt like very connected to what was told to me was gender. But like [it was] probably around when I started doing swim team and I couldn't wear the swimming trunks like the boys could, which was probably maybe around like seven‐ish or six (Drew, Nonbinary/Genderqueer, 24).

Some participants (*n* = 5; 3 nonbinary/genderqueer; 1 transgender man; 1 transmasculine) described knowing they were not like other people who shared their assigned gender but attempting to reject those feelings and/or closely conforming to or identifying with societal expectations related to their assigned gender. One participant who identified within the gender binary also noted this experience.

I threw myself into trying to make myself as pretty as I possibly could. I watched tutorial after tutorial to do my makeup. I taught myself to do my nails. I mean I put on a really good show, but I was dying on the inside, and I didn't know what was going on (Andrew, Transgender Man, 41).

Going into puberty I was like always that boy who was cross‐dressing and, you know, I grew up in the South and that was really difficult, so I definitely repressed any kind of idea around non‐conformity to a binary. Until I was 28, I just tried to live as a man (Avery, Nonbinary/Genderqueer, 29).

Some participants (*n* = 4; 2 transmasculine; 1 transgender man; 1 nonbinary/genderqueer) noted that puberty onset was an initial indicator they were not their gender assigned at birth. Among this group, three participants were assigned female at birth and one participant was assigned male at birth. Participants also noted that people's response to their distress surrounding puberty normalized their reactions as typical, thus potentially delaying their identity exploration or recognition at that time.

I was pretty young when puberty hit like I was nine or 10… at that age anybody might be a little distressed by it, so I think my mom just kind of wrote it off as like, ‘hey you are a little young like this might not feel good right now, but like as you grow up this will feel better’ and it never did (Kai, Transmasculine, 30).

I started having questions about my gender, or rather I really started to hate my body when it decided to go through puberty…And it took a long time to figure out it was a gender issue because I was told ‘Oh yeah all girls hate their body’, so it's just like, ‘You just hate your body like all the other girls’. But [that] definitely set me back at that point in time (Leo, Transgender Man, 23).

The majority of participants (*n* = 12; 4 nonbinary/genderqueer; 1 transmasculine; 5 transgender men; 2 transgender women) described their sexual orientation as impacting how they thought about, explored or contextualized their gender identity development. Of note, no participants described puberty and sexual orientation as collectively impacting their gender identity development.

I was getting stuck on gender and sexuality together and that not necessarily being something that I wanted. It was a very compulsive heterosexuality, that's what I was tripping up on. I want to be a woman, but I don't like men and I realized later, ‘wait, I can just be gay and trans’, and from there I was like yeah I'm trans and never once questioned it again (Emma, Transgender Women, 22).

I guess, a lot of my gender identity has been shaped by like accepting its interaction with my sexual identity… There's definitely just a process of self‐realization and self‐acceptance. But it did feel strange to want to be attracted to women, and also be a woman (Alice, Transgender Women, 28).

I did not know [what] being nonbinary was until I was probably 16 or 17. At that time I was still very much coming to terms with sexuality, so I think it kind of took a back burner for quite a while … I started using like, she/they pronouns for a while, just to feel it out, and never really understood why anyone else identified as any gender they were assigned. That was always quite confusing to me. It's like, ‘Okay, but why? I never felt that way’. I just thought that's how everybody felt (Sage, Nonbinary/Genderqueer, 23).

### Contextual and social influences on gender identity journey

Participants described their context as vital to their gender identity journey. All referenced online contexts as an important place to access their community and resources (*N* = 15) and many referenced the role of geographical or cultural contexts (*n* = 9; one transmasculine; three transgender men; one transgender woman; four nonbinary/genderqueer) in their ability to access information about being TGN. Participants described how growing up in regions and cultures where people were generally less accepting or less aware of TGN identities hindered their identity exploration, and so they were often not exposed to TGN information except online (e.g. YouTube transition timelines and online communities of other TGN people) or in specific contexts (e.g. gender–sexuality alliance (GSA) in school).

I grew up in, like a really conservative, like Christian environment, …in the South in the late eighties, early nineties. That [being TGN] was just not a thing that anybody talked about and so I didn't have the language for it…And then at some point, I just came across it online, and it was like, Oh, man, this is an actual option. People actually do this like what the heck, and it was just like an avalanche of like, holy crap, this all makes sense now… And once I had that word it was just like everything clicked, and then I just kind of ran with it (Daniel, Transgender Man, 34 years old).

For most participants (*n* = 14; 5 nonbinary/genderqueer; 2 transmasculine; 2 transgender women; 5 transgender men), simple exposure or lack thereof to TGN information played a large role in their gender identity journey. In particular, the timing in which they were able to put a name or label to their identities. Many participants, especially those who indicated they grew up in conservative areas of the United States, shared that online resources were critical to their identity recognition.

There's definitely like, creators [online] who are just really open about their stories [of being TGN] and like, it was just listening to those and being like, ‘Oh, that hits. Yikes’. And then like, eventually figuring it out with time like, the more you hear the more you're like, ‘Oh that's what that was’ kind of thing (Sage, Nonbinary/Genderqueer, 23).

Many participants (*n* = 9; 5 nonbinary/genderqueer; 2 transmasculine; 2 transgender men) touched on educational contexts as a safe space for them to explore their gender identity. For example, several participants mentioned their experiences with other TGN students: being asked their pronouns and trying new pronouns, or exposure to ideas of gender outside of the binary. These experiences highlight the linkage of context (e.g. school) and social influences (e.g. friends in school). Such exposure led participants to think about their identity more intentionally than they had previously.

In high school I had friends who had come out as trans, people I had known since elementary school and that was my first introduction to transness and I felt drawn to these people in a way I couldn't really explain. I was [also] part of the high school GSA and so trans people were there, people who were nonbinary, which was a new thing that I learned about at that point. Then my senior year of high school, only in GSA, I said oh I'll use she/her and they/them pronoun… And [later] a lot of people would just automatically use she/her which, like, I found myself, reflecting on like ‘this is a little distressing, why is that?’ And then, when people would use they/them pronouns I felt really affirmed (Drew, Nonbinary/Genderqueer, 24).

All participants (*N* = 15) discussed social influences on their gender identity journey with most citing positive influences (*n* = 12; 4 nonbinary/genderqueer; 2 transmasculine; 4 transgender men; 2 transgender women). These positive social influences primarily came from friends, especially other people in the community.

I knew a nonbinary person in high school, and we have very similar identity sets, so it was like, ‘Oh, that's an option’ kind of thing. Anyone who you know like, gave me opportunities for education and to just kind of learn more I think were really influential (Sage, Nonbinary/Genderqueer, 23).

Some participants also noted their families played a positive role in their gender identity journey.

So I was a kid in the 80s… And growing up in rural [state]… So frankly the best descriptor my folks and I had for me was a tomboy, and it wasn't comfortable, but it was better than my folks calling me a girl or anything like that. I had an astonishingly accepting dad for the times. It was just never any big deal in our house. It was just accepted that, that's how I was, that's who I am– everything (Jack, Transgender Man, 41).

Other positive influences included role models that participants looked to as positive representatives of their own identity. Several participants shared this i.e. anywhere from seeing people's transition journeys online, to seeing themselves in their family members, friends or even teachers. Of note, role models came from many sources and were comprised of both other TGN people and cisgender people whom the participants looked to as ideal representations of some characteristics associated with their gender identity. In particular, 11 participants shared that they had role models from within the TGN community (i.e. 2 transgender women, 2 transgender men, 5 nonbinary/genderqueer and 2 transmasculine) and 9 noted that they had cisgender role models (i.e. 5 transgender men, 2 nonbinary/genderqueer and 2 transmasculine).

One of my teachers in high school… had a very particular type of masculinity that I really appreciated that was not very toxic, but still clearly masculinity that really appealed to me. I don't like the idea of having to be a toxic man in order to pass as a man, so I really sought out forms of masculinity that did not fall into that category (Leo, Transgender Man, 23).

Although most participants shared the positive influence of others on their identity journey, more than half cited negative influences as well (*n* = 8; 4 transgender men; 3 nonbinary/genderqueer; 1 transmasculine). Participants shared that living in unaccepting social environments and social pressures to conform hindered their exploration of their identities fully, yet they still eventually came to recognize their identities.

When I was a kid I was very masculine and tomboyish, and that was seen as like normal, I guess, for an ‘AFAB’ [assigned female at birth] kid, and then, as I grew older, it started to become a lot less acceptable. At some point, it was mostly [due to] social pressure, I started to be a lot more feminine like high fem, all the time (Noah, Transgender Man, 31).

My parents are both from the Soviet Union, so there was not a whole lot of positive queer talk… I also went to a school that did not have a very inclusive education so I just wasn't exposed to that part of my identity… Being a part of a culture that is not very accepting of queer people is probably something that was hindering when I was figuring out my gender identity earlier on… In spite of that, I am still queer (Ash, Nonbinary/Genderqueer, 23).

Another common theme in negative responses that shaped participants' gender identity journey related to dismissive or invalidating comments made by others.

I feel like the back and forth with my presentation and what I want to present versus do present has been shaped like pretty highly by social interactions. [For example] I really wanted to buy board shorts when I was like a preteen and my mom, she doesn't even remember this, she like got really embarrassed, and said that I look too much like a dyke (Noah, Transgender Man, 31).

## DISCUSSION

This study is an initial exploration of White American TGN adults' retrospection on their gender identity development. While many participants identified moments during their childhood when they began to explore their identity, they also noted that the timing of this exploration was shaped by opportunities, contexts and social influences. Thus, the development of TGN gender identity may reflect greater heterogeneity than the development of gender identity broadly. Although there is diversity in the experiences related to participants' identity development, there are also shared experiences. Most participants described the role their contexts and social groups played in this process, both the good (e.g. being given the room to explore their identity and positive role models) and the bad (e.g. negative perspectives about queer people from others and social pressure to act or dress specific ways). This study provides new insight into key developmental markers for the TGN people's gender identity journey, with particular attention to contextual and social influences.

Although only a brief glimpse into the lives of TGN adults' retrospective accounts of their gender identity journey, this study demonstrates how important contexts and social relationships were during the exploration of their identities. Notably, the role of friends, school context and puberty were especially common. Consistent with findings that close social relationships are important for identity development (Ragelienė, 2016), many participants shared that friendship, especially with other TGN people, played an important role in their gender identity development. Some research documents the important role that relationships with peers play in sexual orientation formation (Callahan & McGuire, 2022). Similarly, it may be that supportive friends offer opportunities for TGN children to explore their gender identity, such as through trying different names and pronouns. Furthermore, exploring one's identity at the same time as a friend in a similar identity exploration process may provide solidarity.

Some participants also noted that members of their families were important for their gender identity journeys, sharing moments when their families acted in ways that supported or hindered their exploration. These experiences are consistent with prior work examining family responses, both positive and negative, to TGN family member's gender identity transitions (Andrzejewski et al., 2021; Hale et al., 2021; McGuire et al., 2016). In instances where TGN youth may have less supportive families, friendships may be especially important for the healthy identity exploration and development of these youth. Prior work documented that during the COVID‐19 pandemic, LGBT+ youth with unsupportive parents felt a loss in in‐person identity‐based socialization (Fish et al., 2020). Through online connections with LGBT+ peers, youth were able to find community and support (Fish et al., 2020). Thus, it may be that when TGN youth are unable to find support during their transition process from families and friends in person, online may act as an important source of community, connectedness and support during their gender identity development. However, additional research is needed to further understand the role of friendships, families and online contexts in TGN gender identity development.

Schools and other social contexts can provide opportunities for TGN youth to find gender role models. These role models can act as positive examples of what being a person of a particular gender can look like or even offer insight into their own experiences with their gender identity development. Many participants shared the positive impact that gender role models had on their gender identity development. Participants shared that they felt drawn to people whom they saw themselves in or whom they admired due to how these models presented themselves. This particular finding aligns with the Social Cognitive Theory (Bandura, 1986; Bussey & Bandura, 1999) of gender development in that participants report that they paid attention to models in whom they saw themselves and integrated that information into their self‐concept. However, it is less clear if they specifically used this information to construct their internalized standards related to what behaviours or characteristics they perceived as appropriate for their own gender identity. Thus, future research may choose to explore an application of Social Cognitive Theory to the gender identity development of TGN youth as they navigate their social worlds and look towards gender role models.

Given the amount of time young people spend in schools, school contexts have important implications for their development (Eccles & Roeser, 2013; Johnson, 2009). For many of the participants in this study, school context played an important role in their gender identity development. Participants indicated both positive and negative ways that their educational context impacted their gender identity development. Schools can provide environments where TGN children feel safe to explore their identity and spend time with peers with similar experiences to themselves, such as within GSAs (Feijo et al., 2022; Marx, 2019). Research on school environment has found that engagement with GSAs is related to reduced mental health concerns and positive development among sexual and gender minority youth (Poteat, Calzo, et al., 2020; Poteat, Rivers, & Vecho, 2020).

School contexts can also negatively impact TGN youth's gender identity development. Educational contexts that exclude information on gender diversity or provide outdated or inaccurate information related to gender diversity may impede the process of TGN children's recognition of their gender identity. Furthermore, gendered policies and practices that maintain cisnormativity in schools (e.g. dress codes and gender‐segregated courses; Woolley, 2019), as well as some current school policies regarding name/pronoun use (Renley et al., 2022) and state laws forcing the outing of transgender youth in schools (Human Rights Campaign, 2023), may further impede this process. Future research should attend to the role of contexts, especially educational contexts, in the gender identity development of TGN youth.

The onset of puberty was an impactful process in TGN gender identity development. For some participants, the distress they experienced related to these changes was an indicator that they were TGN. However, given that puberty is characterized as a time of great distress for all youth (Mendle, 2014), their concerns were dismissed by others. Related to TGN youth in particular, a review on gender identity development during adolescence suggests that puberty for children whose gender identity is not established in childhood may be a crucial factor in their gender identity development (Steensma et al., 2013). Furthermore, some work suggests that gender identity may be more flexible prior to puberty, but that puberty may act as a catalyst for gender identification among TGN youth (Byne et al., 2012). Thus, given prior work as well as the current study, continuing to examine the role of puberty and family's response to puberty in the gender identity development of TGN youth may be an important area for future research.

Another related consideration is the relationship between sexual orientation and gender identity. Our findings indicate that gender identity and sexual orientation may be co‐evolving for some people, but they may not be in others. The majority of participants described how their gender identity development was impacted by their sexual orientation. Participants often described their sexual orientation and its development as intrinsically linked to their gender identity and its development (e.g. Emma described ‘compulsive heterosexuality’ impacting her identity exploration). Some participants described prioritizing an exploration of their sexual orientation over an exploration of their gender identity, whereas others described negotiating the process of both at the same time. Future research should consider exploring how potential barriers to identity exploration, such as compulsive heterosexuality, impact gender identity development.

Although the participants in this sample did not explicitly describe puberty as related to their sexual orientation and gender identity, prior research indicates that sexual orientation and puberty are developmentally related (Ostovich & Sabini, 2005). Other research indicates that sexual orientation and gender identity development among TGN youth are related (Callahan & McGuire, 2022). Furthermore, a recent review of sexual orientation and gender identity demonstrated the importance of considering the intersectionality of gender identity and sexual orientation within the developmental sciences (Baams & Kaufman, 2023). Thus, it is likely that puberty and sexual orientation are collectively influential for gender identity development among at least some TGN youth. Scholars should continue to consider the potential developmental implications of puberty and sexual orientation exploration on gender identity development among TGN youth.

Notably, many participants highlighted that given the time and place where they grew up and the social norms of the communities where they were rai.e. they did not recognize other options for their gender identity beyond the identity they were assigned at birth. Yet, even within this, a number of participants described recognizing that their assigned gender did not fit how they saw themselves, even before they had words to be able to describe their identity. The time between these two acknowledgements and what factors may impact the timing between these two acknowledgements may be an interesting area for future exploration. Furthermore, these findings may extend upon cognitive theories of gender development (Kohlberg, 1966; Liben & Bigler, 2002; Martin & Halverson, 1981; Martin & Ruble, 2004). In particular, findings from this study may provide a more nuanced view of self‐socialization among TGN people. Following a recognition that their assigned gender did not fit them, some participants described how they sought out information to understand their feelings, and they found gender role models that demonstrated possibilities they were unaware of, as previously mentioned. Future research should consider exploring how TGN people seek out gender identity information when exploring their gender identity as something other than their assigned gender, and how they apply that information to their gender identities and self‐concepts (i.e. gender self‐socialization). It may be especially interesting to explore this process within the period between acknowledging that their identity does not fit with their assigned gender and coming to acknowledge their specific identity.

Related to this, participants noted online spaces were a key sociali.e. where they were able to interact with gender role models as well as think about and explore possibilities that may not have been available in their immediate community. This finding is similar to other research that documents the importance of online community and support for LGBT+ youth (Fish et al., 2020). Thus, while gender identity development often begins quite early in childhood (Leaper & Bigler, 2011; Leman & Tenenbaum, 2013; Ruble et al., 2006), the findings of the current study suggest that for TGN people, gender identity development may begin and progress at different times for individuals depending on their access to knowledge around gender diversity, their opportunity for self‐education online, their social and family network and their community and school contexts.

### Limitations and future directions

The current study demonstrated great heterogeneity in TGN people's gender identity journey, although participants consistently highlighted the role of social and contextual factors in their experiences. However, these findings are not generalizable or conclusive, as is the case with any qualitative study. Furthermore, these findings may not reflect the experiences of TGN people of colour or those outside of the United States, as the sample was comprised of White TGN individuals within the United States.

Although retrospective approaches are important given the challenges of working with TGN youth, an additional limitation is the retrospective study design, as research documents retrospective accounts of development may be vulnerable to recall bias or other limitations that may influence the accuracy of participants' responses (Hegarty, 2009). When it comes to the types of events people recall, adults are more likely to recall events earlier in their lives tied to strong emotions, such as joy and fear, with most memories occurring after the age of 5 (Pillemer & White, 1989). In terms of accuracy, some research indicates that people are more likely to overestimate the positive impact of past emotionally positive events but they are more accurate in assessing past emotionally negative events and events with more personal relevance, but this accuracy varies both within and between people (Ottenstein & Lischetzke, 2020). Thus, future research should consider not only the type of emotions associated with the recalled memory but the age of the memory and its personal relevance to attempt to assess the accuracy of the recollection.

Another limitation is that many participants identified outside of the binary, so the findings may be less generalizable to binary transgender individuals. However, this could also be considered a strength, given much of prior research has primarily considered the experiences of binary transgender individuals (Veale et al., 2022). Future research may consider intentionally addressing the differential experiences between TGN people who identify inside and outside of the gender binary in their gender identity development and the role of social and contextual factors in this process. Through more inclusive research on the experiences of TGN people's gender identity journey and their experiences with gender diversity, we will be better able to understand not only TGN people's gender identity development but also attain a more holistic understanding of gender identity development. Finally, it should be noted that this is only a small look into the social and contextual factors that are related to gender identity development for a subset of the TGN community. Thus, future research should continue to examine the role of social and contextual factors in the gender identity development of TGN people.

## CONCLUSION

This study provides new insight into how TGN adults retrospectively consider the role of their social environment and context in their gender identity development. However, to truly understand the complexities of gender identity development, much more work needs to be done. It is important we continue to explore TGN people's gender identity development as a diverse process, paying close attention to their social relationships, contexts and the interplay of these factors on their identity exploration and development. Through this continued research we will gain a more complete understanding of gender identity development for TGN people.

## AUTHOR CONTRIBUTIONS

**Emily Herry:** Conceptualization; investigation; formal analysis; funding acquisition; writing – original draft; methodology; supervision; writing – review and editing. **S. M. Rodan:** Data curation; writing – review and editing. **Madeline Martin:** Data curation; writing – review and editing. **Mariam M. Sanjak:** Data curation; writing – review and editing. **Kelly Lynn Mulvey:** Supervision; writing – review and editing.

## CONFLICT OF INTEREST STATEMENT

The authors have no financial or non‐financial conflicts of interest regarding the materials or subject matter discussed in this manuscript.

## Data Availability

The data that support the findings of this study are not shared. The data are not publicly available due to privacy or ethical restrictions.
